# Myocardial T1 and ECV mapping: how we optimise technical aspects of acquisition

**DOI:** 10.1186/1532-429X-18-S1-T9

**Published:** 2016-01-27

**Authors:** Ricardo Wage, Peter Gatehouse, Nur Hayati Jasmin, Dudley J Pennell

**Affiliations:** grid.439338.6Royal Brompton Hospital, London, United Kingdom

## Background

MOLLI techniques for the acquisition of T1 maps in the heart are becoming routine practice at many centres. A wide range of techniques and scanning protocols exist in order to produce reproducible and accurate T1 maps [review in Radiographics, consensus paper James Moon]. However, familiarity and experience using these protocols may be limited outside dedicated CMR units.

Our aim is to present practical experience with a specific T1/ECV scanning protocol. We do not intend to suggest that this or any other protocol is the "correct" one, as this is subject to much ongoing investigation.

## Methods

This work is based on the Siemens prototype WIP 448B and its default protocols for 11 heartbeat variants of MOLLI that have been optimized for typical pre-Gad and post-Gad ranges of T1 values. We present a mixture of experience at 1.5T and 3T.

*Step 1 : Retrospective-gated TruFISP 2D cine of the Mid-LV sax. The retro-gating is quite important in this application to see the diastolic pause in full.*

*Step 2: By viewing the cine with the trigger-time of each frame, find the LV mid-diastolic timing in milliseconds after R-wave trigger, it may be beneficial to optimise timings for septal stability.*

*Step 3 : Select the T1 mapping protocol optimised for longer T1 values pre-Gad. If diastolic pause is short, select a lower-resolution version which has a shorter shot duration. Adjust MOLLI acquisition timing to match image acquisition into the middle of the diastolic pause.*

*Step 4: Set the "adjustments volume" (green box) over the LV (approx 12 × 12 × 12 cm cube). [on VB17 this is normally only a reference frequency adjust not a cardiac shim, use only cardiac shim if available. The "standard" or "advanced" Siemens shim modes are unreliable for cardiac work.*

*Step 5: Acquire the MOLLI image in Breath-hold 11 cardiac cycles. Monitor ECG during this scan and record any abnormal cardiac cycles or triggers.*

*Step 6: Image review: This should be performed rigorously during scanning in case re-acquisition is necessary.*

*Step 7: For Post Contrast study after 14-15 mins of injection.(with altered T1mapping protocol for improved precision in the short-T1 range expected post-Gad). Ensure slice location and timing of shot acquisition are consistent with pre-Gad and post Gad.*

## Results

A detailed presentation of one centre's experience with T1/ECV mapping in a variety of challenging patients. Particular emphasis is placed on acquisition as a major pitfall exists that a T1Map is automatically generated from any input images but should be avoided for T1 measurements without reviewing the underlying image quality.

## Conclusions

Familiarity and experience with T1 Mapping may be limited outside of high volume CMR units. Detailed practical guidance to minimize the number of non-diagnostic scans may reduce the "scatter" in T1 and ECV measurements, which is currently of large concern for applying these techniques.

**Figure 1:**
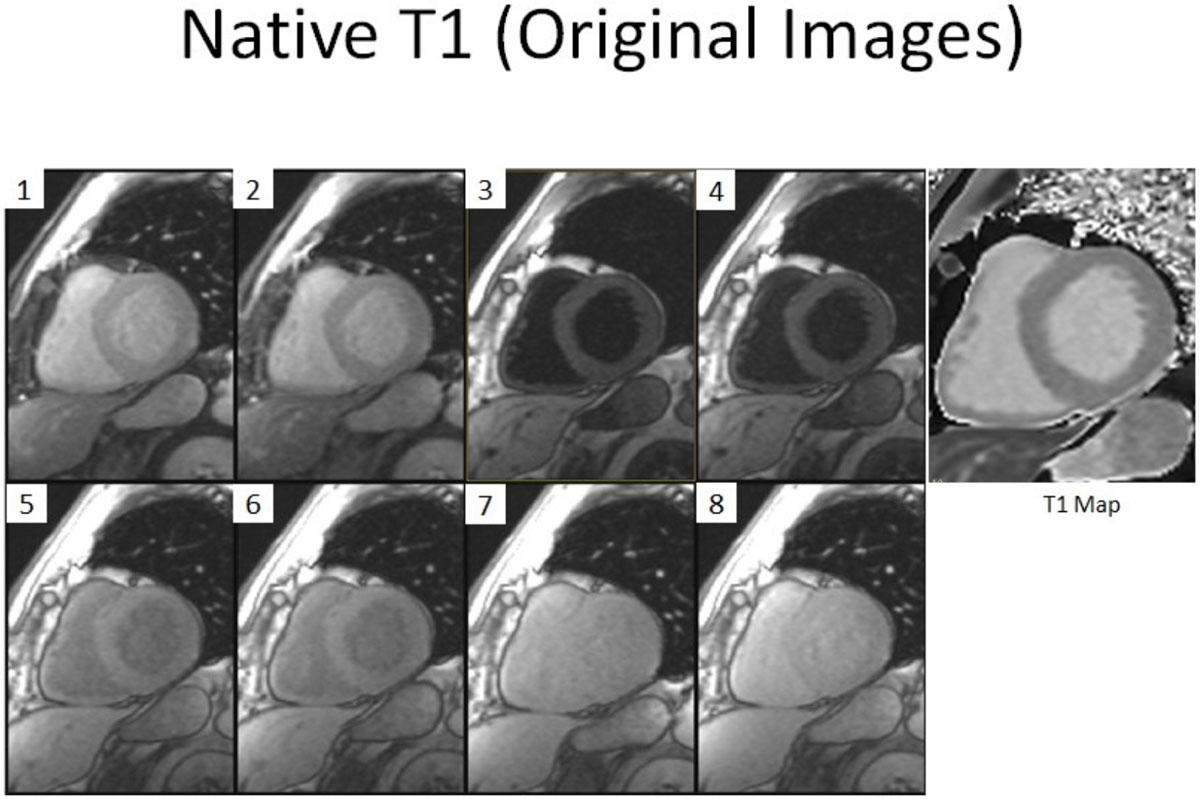
Figure 1

**Figure 2:**
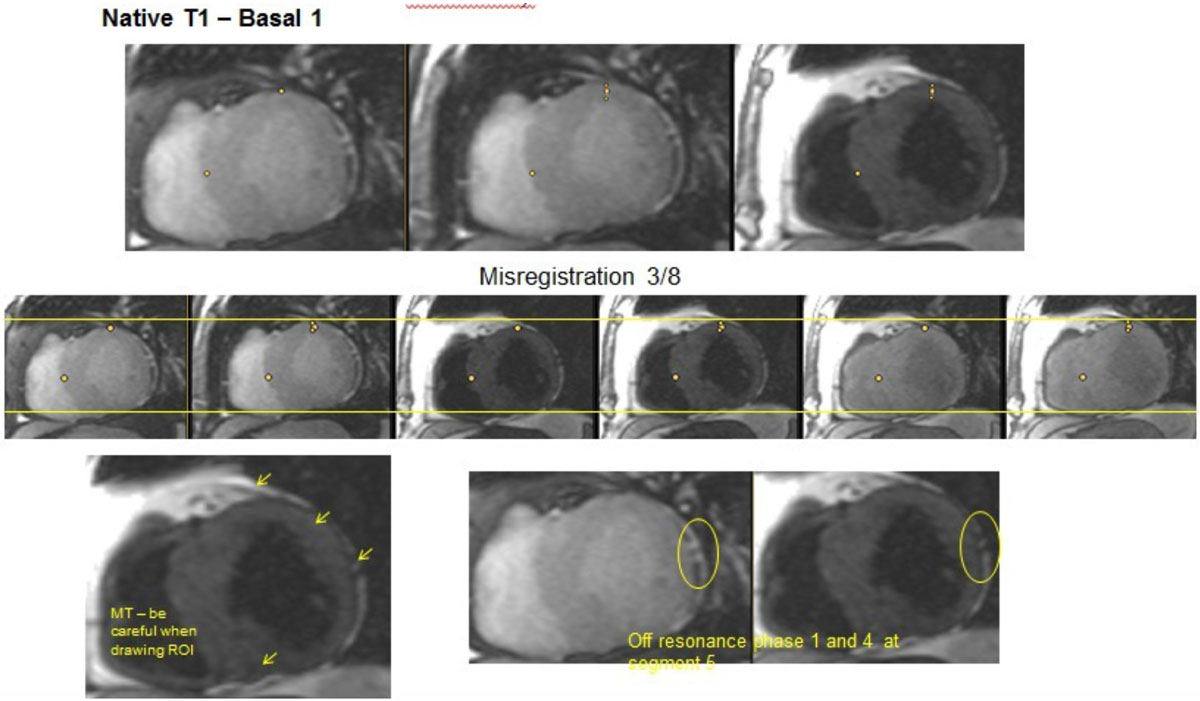
Figure 2

